# Clinical value of a prophylactic minitracheostomy after esophagectomy: analysis in patients at high risk for postoperative pulmonary complications

**DOI:** 10.1186/s12893-017-0321-z

**Published:** 2017-12-01

**Authors:** Yayoi Sakatoku, Masahide Fukaya, Kazushi Miyata, Keita Itatsu, Masato Nagino

**Affiliations:** Division of Surgical Oncology, Department of Surgery, Nagoya University Graduate School of Medicine, 65 Tsurumai-cho, Showa-ku, Nagoya, 466-8550 Japan

**Keywords:** Minitracheostomy, Postoperative pneumonia, Esophagectomy

## Abstract

**Background:**

The aim of this study is to evaluate the clinical value of a prophylactic minitracheostomy (PMT) in patients undergoing an esophagectomy for esophageal cancer and to clarify the indications for a PMT.

**Methods:**

Ninety-four patients who underwent right transthoracic esophagectomy for esophageal cancer between January 2009 and December 2013 were studied. Short surgical outcomes were retrospectively compared between 30 patients at high risk for postoperative pulmonary complications who underwent a PMT (PMT group) and 64 patients at standard risk without a PMT (non-PMT group). Furthermore, 12 patients who required a delayed minitracheostomy (DMT) due to postoperative sputum retention were reviewed in detail, and risk factors related to a DMT were also analyzed to assess the indications for a PMT.

**Results:**

Preoperative pulmonary function was lower in the PMT group than in the non-PMT group: FEV1.0 (2.41 vs. 2.68 L, *p* = 0.035), and the proportion of patients with FEV1.0% <60 (13.3% vs. 0%, *p* = 0.009). No between-group differences were observed in the proportion of patients who suffered from postoperative pneumonia, atelectasis, or re-intubation due to respiratory failure. Of the 12 patients with a DMT, 11 developed postoperative pneumonia, and three required re-intubation due to severe pneumonia. Multivariate analysis revealed FEV1.0% <70% and vocal cord palsy were independent risk factors related to a DMT.

**Conclusion:**

A PMT for high-risk patients may prevent an increase in the incidence of postoperative pneumonia and re-intubation. The PMT indications should be expanded for patients with vocal cord palsy or mild obstructive respiratory disturbances.

## Background

In Japan, the standard surgical procedure for esophageal cancer is subtotal esophagectomy with extended lymph node dissection, which requires the skeletonization of the upper mediastinal structures. This procedure is highly invasive, with high morbidity and mortality rates [1, 2]. Postoperative pneumonia is the most serious complication after esophagectomy and is a major risk factor for in-hospital mortality [3]. Impairment of the swallowing function due to cervical lymph node dissection and vocal cord palsy resulting from para-laryngeal nerve lymph node dissection both cause pulmonary aspiration. The impairment of postoperative pulmonary function and postoperative chest pain induce difficulty in expectoration, which can lead to sputum retention and postoperative pneumonia.

Although bronchoscopic aspiration is typically performed for sputum retention, this procedure requires trained bronchoscopists; a significant delay often occurs from onset to treatment. Bronchoscopic aspiration places a large burden on patients. Local anesthetic administered to the mucous membranes of the pharynx, larynx, and trachea often induces the pulmonary aspiration of intraoral bacteria. However, a minitracheostomy allows nursing staff without specialized training to have immediate access to the bronchial tree. The introduction of a catheter into the trachea through the minitracheostomy typically evokes an effective cough that helps clear secretions.

Previous authors have reported that a prophylactic minitracheostomy (PMT) helps prevent postoperative pulmonary complications in patients who undergo pulmonary resection for lung cancer [4–8]. However, given the limited number of available reports, the clinical value of a PMT is unclear in patients undergoing an esophagectomy. Since January 2009, we have used a PMT in patients at a high risk for postoperative pulmonary complications to decrease these complications. The aim of this study was to evaluate the clinical value of a PMT in patients undergoing an esophagectomy for esophageal cancer and to clarify the indications for a PMT.

## M**ethods**

### Patients

From January 2009 to December 2013, 99 patients underwent a right transthoracic esophagectomy via muscle sparing thoracotomy (MST) as reported previously [9]. Of these, two patients with a previous laryngectomy and one patient with a synchronous laryngectomy were excluded. Two other patients who underwent a tracheostomy for delayed extubation were also excluded. Thus, the remaining 94 patients were subjected to analysis. The ethical committee of Nagoya University Hospital approved our study (No. 2016–0361); written informed consent was obtained from all patients.

### Surgical procedures

All patients underwent a right transthoracic esophagectomy via MST with mediastinal lymphadenectomy, including bilateral recurrent laryngeal nerve lymph node dissection and laparotomy for dissecting abdominal lymph nodes, to establish a reconstructive conduit. Thoracotomy was followed by laparotomy in patients with borderline resectable tumors, while laparotomy was followed by thoracotomy in all other patients. The gastric tube was selected as the primary reconstructive conduit. The percutaneous route was chosen in patients who were older or who had liver cirrhosis, and the retrosternal route was used in patients with possible residual tumors (R1/2 resection). In the other patients, the choice of the reconstruction route that was used depended on the surgeon’s preference. Reconstruction with a pedicled jejunum was performed via the percutaneous route in all patients who had previously undergone or synchronously underwent gastrectomy.

### Prophylactic minitracheostomy

The tracheal tube was routinely extubated on the first postoperative day if the general condition of the patients was stable. The degree of vocal cord palsy was evaluated by bronchoscopy in all patients just after extubation, and a PMT was subsequently performed using a Minitrach II® (SIMS Portex, Hythe, Kent, UK) with the percutaneous Seldinger technique for patients at high risk of postoperative pulmonary complications. These patients included elderly patients over 80 years of age, patients with vocal cord palsy and the presence of a slit between the vocal cords, patients with low pulmonary function [(a forced expiratory volume in 1 s (FEV1.0) <1.5 L or a percent predicted forced expiratory volume in 1 s (FEV1.0%) <60%)], patients with preoperative pneumonia, including interstitial pneumonia, and patients with aspiration noted in an upper gastrointestinal image (Table 1). Routine prophylactic aspiration by bronchoscopy was never performed. A mini-tracheal tube was extubated unless the patients developed pulmonary aspiration after the start of oral intake. A total of 30 patients underwent a PMT; 16 patients were selected to undergo a PMT before surgery, and the remaining 14 patients underwent a PMT after surgery. We performed a delayed minitracheostomy (DMT) following bronchoscopic aspiration for patients with postoperative sputum retention despite the presence of vocal cord palsy.

**Table 1:** Indication of prophylactic minitracheostomy

Indication	Number of patients
Preoperative	
Old age	2
Low pulmonary function	5
Preoperative pneumonia	5
Aspiration	3
Low pulmonary function + Aspiration	1
Postoperative	
Vocal cord palsy	14

### Perioperative care

All patients received intravenous injections of methylprednisolone to attenuate the inflammatory responses as follows: 250 mg intravenously 1 h before the start of surgery, 125 mg on day 1, and 80 mg on day 2. One epidural catheter was intubated between the fifth and sixth thoracic vertebra, and another epidural catheter was intubated between the ninth and tenth thoracic vertebra. Continuous epidural anesthesia with fentanyl and ropivacaine or levobupivacaine was used until day 6. An intravenous drip injection of pentazocine (15 mg) or buprenorphine (3 mg) was administered as needed until day 10. An injection of loxoprofen or pregabalin was administered via feeding tube from day 11 until the start of oral intake. Computed tomography (CT) was performed on day 7 in all patients. Atelectasis was assessed by radiological evidence of plate atelectasis, labor collapse, or total lung collapse as shown on the CT image.

Postoperative complications were defined as any event requiring specific medical or surgical treatment and were assessed according to the Clavien-Dindo classification [10]. A PMT was not considered to be a grade 3 pulmonary complication.

### Statistical analyses

The results are expressed as the median (range). Fisher’s exact probability test and the Mann-Whitney *U* test were used for analysis as appropriate. Univariate and multivariate analyses were performed using a logistic regression model to identify the independent factors that were associated with postoperative pneumonia. In the multivariate analysis, the factors that showed a *p* value of <0.200 in the univariate analysis were selected and subjected to a stepwise logistic regression analysis. All statistical analyses were performed with SPSS software version 20.0 J. The two-sided *p* values were calculated and are presented. A *p* value of <0.05 was considered statistically significant.

## Results

### Patient characteristics

No significant differences were observed between the PMT and non-PMT groups in terms of the age, gender, tumor location, clinical stage, or proportion of patients who underwent preoperative chemotherapy, preoperative chemoradiotherapy, or a salvage operation (Table 2). Regarding the preoperative pulmonary function, the FEV1.0 was significantly lower in the PMT group than that in the non-PMT group. The proportion of patients with FEV1.0% less than 60% was significantly higher in the PMT group than that in the non-PMT group.

**Table 2:** Patients’ characteristics

Variables	PMT group (*n* = 30)	Non-PMT group (*n* = 64)	*P*
Age [year]	68.5 (51–86)	65.0 (43–78)	0.071
Gender (male/female)	23/7	57/7	0.131
Location of tumor, n (%)			0.413
Ut	6 (20.0)	6 (9.4)	
Mt	13 (43.3)	36 (56.2)	
Lt	9 (30.3)	16 (25.0)	
Ae	2 (6.7)	6 (9.4)	
cStage (UICC 7th), n (%)			0.153
I	5 (16.7)	25 (39.1)	
II	10 (33.3)	14 (21.9)	
III	12 (40.0)	20 (31.2)	
IV	3 (10.0)	5 (7.8)	
Neoadjuvant chemotherapy, n (%)	14 (46.7)	26 (40.6)	0.652
Neoadjuvant chemoradiotherapy, n (%)	6 (20.0)	5 (7.8)	0.099
Salvage operation, n (%)	1 (3.3)	4 (6.3)	1.000
Preoperative pulmonary function			
VC [L]	3.51 (2.03–5.43)	3.66 (2.05–5.57)	0.113
%VC	114 (67–134)	110 (76–166)	0.703
FEV1.0 [L]	2.41 (1.11–3.36)	2.68 (1.59–4.13)	0.035
FEV1.0%	71.5 (53.2–91.3)	76.4 (60.2–93.8)	0.160
FEV1.0% < 60%, n (%)	4 (13.3)	0	0.009

*PMT* prophylactic minitracheostomy

### Surgical procedures

The surgical procedures are summarized in Table 3. No between-group differences were observed in the proportion of patients requiring cervical lymph node dissection, a reconstructive organ, a reconstructive route, and an anastomotic portion. The operative time and blood loss were similar between the two groups.

**Table 3:** Surgical procedures

Variables	PMT group (*n* = 30)	Non-PMT group (*n* = 64)	*P*
Cervical lymph node dissection, n (%)	24 (80.0)	52 (81.3)	1.000
Reconstructed organ, n (%)			0.064
Stomach	27 (90.0)	46 (71.9)	
Jejunum	3 (10.0)	18 (28.1)	
Reconstructive route, n (%)			0.229
Percutaneous	7 (23.4)	20 (31.2)	
Retrosternal	10 (33.3)	11 (17.2)	
Postmediastinal	13 (43.3)	33 (51.6)	
Anastomotic portion			
Cervical / Intrathoracic	23 / 7	40 / 24	0.240
Operative time [min]	540 (406–732)	584 (306–975)	0.084
Blood loss [ml]	1057 (262–2567)	964 (269–6698)	0.320
Blood transfusion, n (%)	19 (63.3)	33 (51.6)	0.374

*PMT* prophylactic minitracheostomy

### Postoperative outcomes

The duration of intubation was significantly longer in the PMT group than in the non-PMT group (Table 4). No significant differences were observed between the two groups in terms of the incidence of grade 2 postoperative pneumonia and atelectasis. Of the 64 non-PMT patients, 12 patients required a DMT due to postoperative sputum retention, and seven required re-intubation. The incidence of vocal cord palsy was significantly higher in the PMT group than that in the non-PMT group because a PMT was performed for patients with vocal cord palsy and the presence of a slit between the vocal cords. No between-group differences were observed in terms of paroxysmal tachycardia or anastomotic leakage. The lengths of postoperative hospital stays were not different. One patient died of severe pneumonia on day 34 in the non-PMT group.

**Table 4:** Postoperative outcomes

Variables	PMT group (*n* = 30)	Non-PMT group (*n* = 64)	*P*
Extubation of tracheal tube [POD]	2 (1–6)	1 (1–11)	0.002
Pulmonary complications, n (%)			
Postoperative pneumonia (≧CD2)	8 (26.7)	25 (39.1)	0.258
Atelectasis^a^	10 (33.3)	26 (40.6)	0.495
Re-intubation	0	7 (10.9)	0.093
Other complications, n (%)			
Vocal cord palsy	16 (53.3)	12 (18.8)	0.001
Paroxysmal tachycardia	7 (23.3)	13 (20.3)	0.790
Anastomotic leakage	0	8 (12.5)	0.052
Any complication (≧CD3a), n (%)	5 (16.7)	21 (32.8)	0.137
90-day mortality, n (%)	0	1 (1.6)	1.000
Postoperative hospital day [days]	28 (16–97)	30 (14–226)	0.460

*PMT* prophylactic minitracheostomy, *CD* Clavien-Dindo classification

^a^diagnosed by computed tomography

Regarding patients with vocal cord palsy, in two patients who underwent the resection of unilateral recurrent nerve involved in metastatic lymph node, ansa cervicalis-recurrent nerve anastomosis was performed simultaneously. Though the vocal palsy was permanent, they kept relatively good phonating function and swallowing function without aspiration. All the other patients with vocal code palsy recovered conservatively within 6 months after the operation. All patients with postoperative aspiration became orally ingestible by swallowing rehabilitation.

Next, we reviewed in detail the 12 patients who underwent a DMT (Table 5). Of these patients, seven had mild obstructive respiratory disturbances, and five had vocal cord palsy. Co-morbidities with liver cirrhosis, heart failure, failed smoking cessation, and walking difficulty were also found.

**Table 5:** The characteristics of the patients with delayed minitracheostomy

	Age	FEV1.0% < 70	Vocal cord palsy	Others factors
1	60–69			Failure to cease tobacco
2	60–69	〇	〇	
3	60–69	〇		Liver cirrhosis (ICGR15 = 19%)
4	60–69		〇	
5	70–79	〇		Walking difficulty
6	70–79			Heart failure (EF48%)
7	40–49			Failure to control pain
8	70–79	〇	〇	
9	70–79	〇		
10	60–69		〇	Liver cirrhosis (ICGR15 = 25%)
11	60–69	〇		
12	70–79	〇	〇	

*ICGR15* indocyanine green retention time 15 min, *EF* ejection fraction

When the 12 patients with a DMT were compared with the 52 patients without a DMT, significant between-group differences were observed in terms of the following parameters: the incidence of postoperative pneumonia (11/12 vs. 14/42, *p* < 0.001, atelectasis (9/12 vs. 11/52, *p* < 0.001), and postoperative hospital stay [50 (18–137) vs. 24 (14–224) days, *p* = 0.008].

Of the 12 patients who received a DMT, three required re-intubation due to severe pneumonia. However, of the 52 patients without a DMT, four underwent re-intubation. These four patients did not undergo a minitracheostomy before re-intubation due to sudden respiratory failure or acute progressive severe pneumonia.

### Logistic regression analysis of the risk factors related to DMT

The risk factors related to a DMT were analyzed using univariate and multivariate logistic regression analyses in the 64 non-PMT patients (Table 6). Nine possible risk factors were included in the analysis. The dysfunction of other organs was defined as a history of ischemic heart disease or heart failure, cerebrovascular disease, liver cirrhosis (indocyanine green retention time at 15 min >15%), or renal failure (serum creatinine level > 1.5 mg/dl). Among these potential risk factors, multivariate analysis identified FEV1.0% < 70% and vocal cord palsy as independent risk factors.

**Table 6:** Uni-and multivariate analyses for risk factors related to delayed mini-tracheostomy

Variables		DMT n (%)	Univariate	Multivariate	
	n		*P*	HR (95%-CI)	*P*
Age			0.238		
75>	55	9 (16.4)			
≧75	9	3 (33.3)			
Brinkman Index			0.968		
800>	37	7 (18.9)			
≧800	27	5 (18.5)			
FEV1.0%			0.061		0.032
≧70	42	5 (11.9)		1	
< 70	22	7 (31.8)		5.06 (1.15–22.21)	
Clinical stage (UICC 7th)			0.838		
I	25	5 (20.0)			
II III IV	39	7 (17.9)			
Preoperative chemoradiotherapy			0.533		
Absent	55	11 (20.0)			
Present	9	1 (11.1)			
Cervical lymph node dissection			0.162		
Absent	12	4 (33.3)			
Present	52	8 (15.4)			
Reconstructive organs			0.790		
Stomach	46	9 (19.6)			
Jejunum	18	3 (16.7)			
Vocal cord palsy			0.032		0.017
Absent	52	7 (13.5)		1	
Present	12	5 (41.7)		6.90 (1.41–33.85)	
Dysfunction of other organs			0.073		
Absent	54	8 (14.8)			
Present	10	4 (40.0)			

*DMT* delayed mini-tracheostomy, *HR* hazard ratio, *CI* confidence interval

## Discussion

Our results demonstrated that the incidence of postoperative pulmonary complications in high-risk patients (the PMT group) was at least equivalent to that in the standard-risk patients (the non-PMT group). A noteworthy observation was that no patient required re-intubation in the PMT group. A PMT may prevent an increase in the incidence of postoperative pneumonia and re-intubation in patients at high risk for pulmonary complications. Although no complications related to a PMT were reported in this study, severe complications associated with a minitracheostomy have been reported, such as membranous tracheal injury, bleeding from the anterior cervical vein, hoarseness, and obstructive subglottic granuloma after removal of a minitracheostomy tube [11–13]. A minitracheostomy may prevent elevation of the larynx during swallowing and impair the swallowing function. Therefore, a PMT should be restricted to high-risk patients, and it is important to appropriately select patients requiring a PMT.

Regarding our PMT indications in this study, age, low pulmonary function, and vocal cord palsy were reported to be associated with postoperative pneumonia after esophagectomy [14, 15]. Aspiration of oral bacteria is commonly known to cause postoperative pneumonia [16]. Vocal cord palsy with a slit and reduced swallowing function with aspiration on the upper gastrointestinal image were therefore included as indications for a PMT. In patients with preoperative pneumonia including interstitial pneumonia, worsening of this condition due to an esophagectomy can be lethal; thus, preoperative pneumonia was also included as an indication for PMT.

A routine tracheostomy may be safer than a minitracheostomy when emergency airway management is needed. However, a tracheostomy leads to temporary voicelessness, which is stressful for patients and causes impairment of the swallowing function due to the restriction of the elevation movement of the larynx during swallowing. Moreover, a tracheostomy can occasionally cause severe complications such as recurrent laryngeal nerve injury, tracheoesophageal fistula, or tracheo-brachiocephalic artery fistula. We propose that a prophylactic tracheostomy is too invasive.

In this study, none of the 30 patients who received a PMT according to our indications required re-intubation, whereas 12 of the non-PMT patients required a DMT due to postoperative sputum retention, and three developed severe pneumonia and required re-intubation. A multivariate analysis revealed that FEV1.0% <70% and vocal cord palsy were independent risk factors related to a DMT. Therefore, the indications for a PMT should be expanded for such patients despite the presence of a slit between the vocal cords. After this analysis, we expanded the indications for a PMT.

Although we focused on pulmonary function and aspiration to define the indications for a PMT, the DMT group included patients with health problems other than pulmonary function, such as liver cirrhosis, heart failure, and walking difficulty. In the prospective randomized trial reported by Pramod et al. [6, 17], the indications for a PMT included ischemic heart disease and cerebrovascular disease, which are likely to be exacerbated by postoperative hypoxia. In their study, some patients developed acute myocardial infarction or cerebellar infarction secondary to sputum retention. In addition to pulmonary function and aspiration, other organ disorders, such as heart failure, ischemic heart disease, liver cirrhosis, cerebrovascular disease, and performance status should be considered for a PMT.

In the present study, 11 of the 12 patients with a DMT due to postoperative sputum retention developed postoperative pneumonia, and three patients progressed to severe pneumonia. These observations demonstrate that a DMT after postoperative sputum retention cannot prevent postoperative pneumonia. In patients with sputum retention, oral bacteria may have dripped into the bronchial tree gradually due to postoperative vocal cord palsy and an impairment of swallowing function immediately after extubation. Thus, when sputum retention occurs, a pulmonary infection may have already developed. It is therefore important to prophylactically perform a minitracheostomy.

Some limitations were associated with this study. First, this is a retrospective study with only a small number of patients. Second, most of our patients underwent cervical lymph node dissection which is not generally performed in western country. Cervical lymph node dissection was reported to increase the incidence of vocal cord palsy [1] and impair swallowing function [18], and may lead to the increase of the incidence of postoperative pneumonia. Therefore, our results do not apply to patients without cervical lymph node dissection, and it may be necessary to reconsider the indications for a PMT for patients without cervical lymph node dissection. Third, all study patients underwent an open thoracotomy. The incidence of pulmonary complication in open thoracotomy has been reported to be 12.5 to 39.66% [19]. The incidence of postoperative pneumonia in our study was 35.1%, and not particularly high, compared with open thoracotomy groups in the other studies. However, thoracoscopic esophagectomy has recently become popular and has been reported to reduce pulmonary complications compared to open thoracotomy [19–21]. It may be necessary to reconsider the PMT indications also for patients receiving thoracoscopic esophagectomy. Fourth, because this study did not compare two groups with the same condition, no conclusive results can be drawn from this comparison. A prospective randomized study comparing a PMT group and a non-PMT group of patients at high risk for pulmonary complications is needed.

## Conclusion

A PMT for patients at high risk for postoperative pulmonary complications may be effective for preventing an increase in the incidence of postoperative pneumonia and re-intubation. The indications for a PMT should be expanded for patients with mild obstructive respiratory disturbances or vocal cord palsy despite the presence of a slit between the vocal cords.

## Abbreviations

CT: Computed tomography
DMT: Delayed minitracheostomy
MST: Muscle sparing thoracotomy
PMT: prophylactic minitracheostomy
